# Environmental Ligands of the Aryl Hydrocarbon Receptor in Allergic Rhinitis: Immune and Neuroimmune Mechanisms

**DOI:** 10.3390/ijms27219545

**Published:** 2026-10-26

**Authors:** Yalan Xiong, Jia Han, Zhaoxin Yang, Yongqin Liu, Peiwen Ding, Xinyi Bian, Bohua Yan, Yu Fang

**Affiliations:** School of Clinical Medicine, Chengdu University of Traditional Chinese Medicine, Chengdu 610075, China

**Keywords:** allergic rhinitis, aryl hydrocarbon receptor, histamine, neuroimmunity, mast cells, immunoregulation

## Abstract

Allergic rhinitis (AR) is an immunoglobulin E (IgE)-mediated chronic inflammatory disease. Although type 2 immune responses are central to AR, environmental exposures, epithelial barrier dysfunction, and neuroimmune interactions also contribute to disease development. The aryl hydrocarbon receptor (AhR) is a ligand-activated transcription factor that links environmental signals to immune responses. Elevated AhR expression in peripheral blood mononuclear cells from patients with AR has been associated with clinical severity. In non-AR experimental systems, AhR suppresses histamine-associated interleukin-6 (IL-6) production in macrophages, whereas 2,3,7,8-tetrachlorodibenzo-p-dioxin (TCDD) reduces acetylcholinesterase (AChE) expression and activity in neuronal cells. These findings suggest possible links to nasal inflammation and cholinergic regulation, but their contribution to nasal symptoms in AR remains to be established. In addition, AhR modulates mast-cell, B-cell, and T-cell responses in a ligand- and cell-dependent manner. Overall, AhR may exert protective or pathogenic effects depending on the activating ligand and target cell type, suggesting that its therapeutic potential in AR lies in ligand- and cell-specific modulation.

## 1. Introduction

Allergic rhinitis (AR) is an immunoglobulin E (IgE)-mediated chronic inflammatory disease characterized primarily by nasal itching, congestion, rhinorrhea, frequent sneezing, and tearing, which significantly impair patients’ quality of life as well as work and academic performance [1,2]. Its etiology is complex, involving both genetic susceptibility and exposure to environmental allergens and air pollutants, while its pathogenesis is largely driven by aberrant immune cell activation and the release of multiple inflammatory mediators [3]. Epidemiological studies indicate substantial global variation in AR prevalence, ranging from approximately 5% to 50% [4]. A review of adult population studies reported a median worldwide AR prevalence of 18.1%, with substantial variation across definitions and populations [5], making AR an important public health concern. AR is associated with impaired sleep and psychological burden [6,7] and commonly coexists with asthma [8]. Current management strategies for allergic diseases mainly include patient education and allergen avoidance, conventional pharmacotherapy, allergen-specific immunotherapy, biologic therapies targeting immune pathways, and other adjunctive or emerging treatments [9]. Despite these treatment options, some patients remain inadequately controlled, supporting further investigation of disease mechanisms and therapeutic targets.

The aryl hydrocarbon receptor (AhR) is a prototypical ligand-activated transcription factor initially identified for mediating the toxic effects of environmental pollutants such as dioxins. It is now recognized as a key molecular hub linking environmental exposure with immune responses [10]. AhR responds to structurally diverse exogenous and endogenous ligands, including environmental aromatic compounds and tryptophan-derived metabolites [11,12]. Its downstream effects include the regulation of immune responses and mucosal homeostasis [13]. In experimental models, exposure to fine particulate matter (PM_2.5_) exacerbates nasal epithelial injury through AhR-associated signaling [14]. AhR signaling has been implicated in immune and inflammatory diseases, including allergic disorders, although its effects vary with the ligand and cellular context [15,16]. In patients with AR, increased AhR expression in peripheral blood mononuclear cells has been associated with clinical severity, although this association does not establish a causal role in nasal inflammation [17]. Therefore, further elucidation of AhR-mediated immune regulation in AR is of considerable importance for clarifying disease pathogenesis and identifying potential targeted therapeutic strategies.

Previous reviews have summarized AhR-mediated immune regulation across allergic diseases [15]. Building on this work, the present review focuses on how environmental ligand exposure may influence epithelial and immune responses in AR through AhR signaling. Findings involving endogenous and synthetic ligands are also discussed to help explain why AhR activation can produce different effects across ligands and cell types. Within this framework, we examine the evidence linking AhR to nasal epithelial injury and immune-cell regulation, together with its proposed involvement in histaminergic and cholinergic pathways relevant to nasal symptoms. Throughout the review, we distinguish findings obtained in AR-related models from those derived from other experimental systems, clarifying which mechanisms are supported by direct evidence and which remain hypotheses requiring validation in AR.

## 2. Biological Functions of AhR and Immune Regulation in AR

AhR belongs to the basic helix–loop–helix PER–ARNT–SIM (bHLH-PAS) transcription factor family and exerts diverse biological functions through binding to both exogenous and endogenous ligands. Its exogenous ligands mainly include halogenated aromatic hydrocarbons (HAHs) and polycyclic aromatic hydrocarbons (PAHs), among which 2,3,7,8-tetrachlorodibenzo-p-dioxin (TCDD), a planar halogenated aromatic toxicant, exhibits extremely high binding affinity for AhR [11]. Endogenous and microbiota-derived AhR ligands include several tryptophan metabolites and indole derivatives [11,12], suggesting that AhR not only participates in sensing exogenous toxicants but also plays important roles in various physiological regulatory processes.

In the absence of ligand binding, AhR remains inactive in the cytoplasm, forming a complex with several chaperone proteins such as heat shock protein 90 (HSP90), the co-chaperone p23, and AhR-interacting protein (AIP) [13]. Upon ligand activation, AhR undergoes conformational changes, dissociates from these chaperones, and translocates into the nucleus, where it forms a heterodimer with the AhR nuclear translocator (ARNT), thereby regulating transcription of multiple target genes including the cytochrome P450 genes *CYP1A1*, *CYP1A2*, and *CYP1B1* [18]. Moreover, AhR signaling is not restricted to the classical AhR/ARNT-dependent transcriptional pathway; it also exerts broad biological effects through interactions with multiple signaling pathways involved in inflammation, immune regulation, and epithelial barrier function [16,18,19].

**Figure 1:**
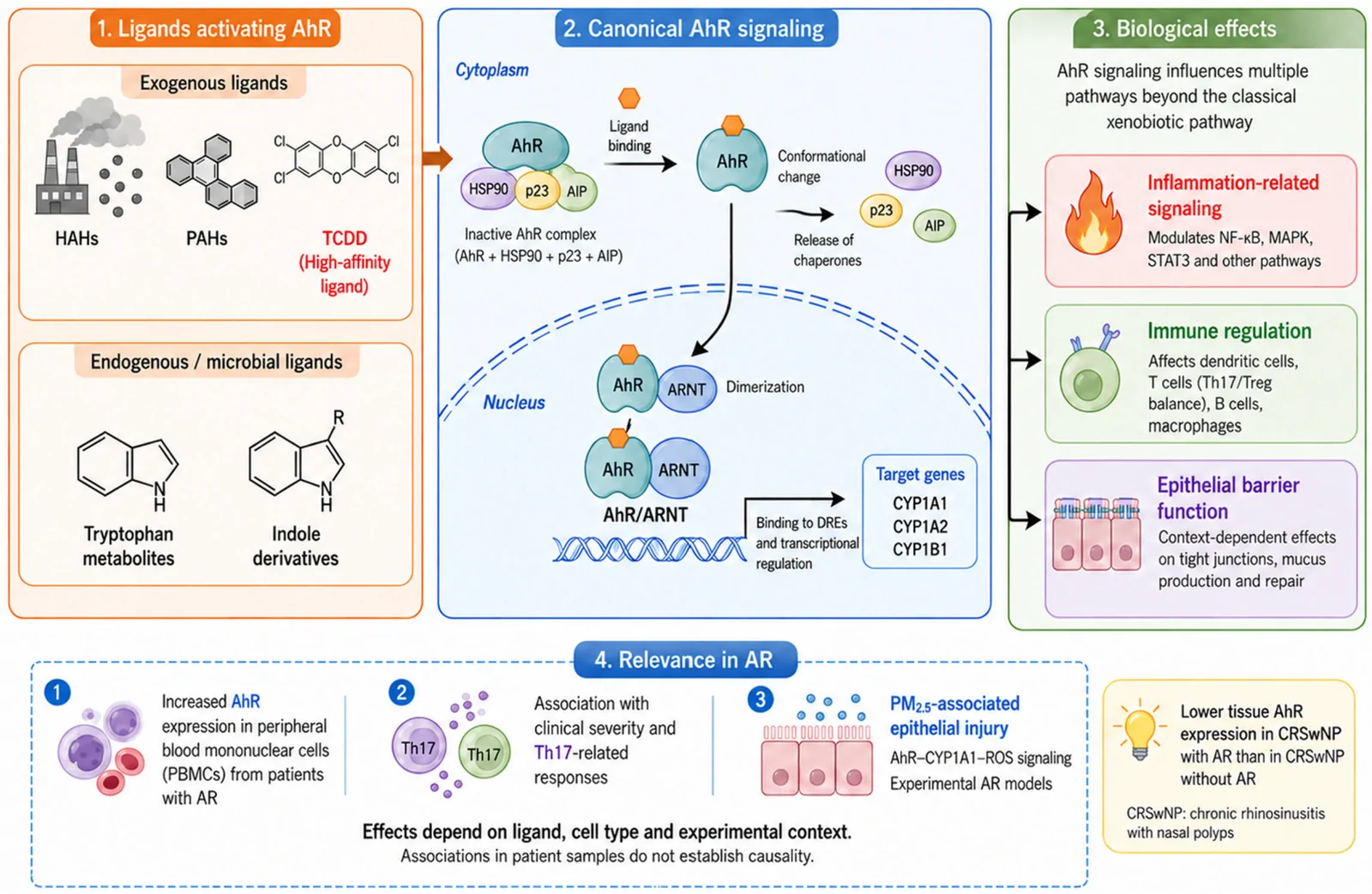
Biological functions of AhR and its relevance to AR. The diagram summarizes exogenous, endogenous, and microbiota-derived ligands; canonical AhR signaling; and context-dependent effects on inflammatory pathways, immune cells, and epithelial barrier function. Following ligand binding and nuclear translocation, AhR heterodimerizes with ARNT and regulates target-gene transcription through DREs. The lower panel distinguishes associations observed in peripheral blood mononuclear cells from experimental evidence of PM_2.5_-associated nasal epithelial injury. The separate inset describes lower tissue AhR expression in CRSwNP with concomitant AR than in CRSwNP without AR; this comparison should not be generalized to isolated AR. Associations in patient samples do not establish causality. Abbreviations: AhR, aryl hydrocarbon receptor; AIP, AhR-interacting protein; AR, allergic rhinitis; ARNT, AhR nuclear translocator; CRSwNP, chronic rhinosinusitis with nasal polyps; *CYP1A1*, *CYP1A2,* and *CYP1B1*, cytochrome P450 1A1, 1A2, and 1B1, respectively; DREs, dioxin response elements; HAHs, halogenated aromatic hydrocarbons; HSP90, heat shock protein 90; MAPK, mitogen-activated protein kinase; NF-κB, nuclear factor κB; PAHs, polycyclic aromatic hydrocarbons; PBMCs, peripheral blood mononuclear cells; PM_2.5_, particulate matter with an aerodynamic diameter of 2.5 μm or less; ROS, reactive oxygen species; STAT3, signal transducer and activator of transcription 3; TCDD, 2,3,7,8-tetrachlorodibenzo-p-dioxin; Th17, T helper 17 cells; Treg, regulatory T cells. p23 denotes the HSP90 co-chaperone p23; R denotes a variable substituent.

Human AR studies include both observational analyses and experiments using patient-derived cells. Increased AhR expression in peripheral blood mononuclear cells has been associated with clinical severity and T helper 17 (Th17)-related responses, but these findings should not be interpreted as direct evidence of increased AhR expression in nasal epithelial cells or of a causal epithelial feedback loop [17]. In patients with chronic rhinosinusitis with nasal polyps (CRSwNP), tissue AhR expression was lower in those with concomitant AR than in those without AR [20]. This comparison concerns distinct CRSwNP subgroups and should not be generalized to isolated AR. However, studies directly elucidating the relationship between AhR signaling pathways and the pathogenesis of AR remain limited. Further studies are needed to define the effects of AhR signaling in specific nasal cell populations and to assess their therapeutic relevance in AR. Figure 1 summarizes canonical AhR signaling and the evidence relevant to AR.

## 3. AhR-Mediated Immune and Neuroimmune Mechanisms in AR

### 3.1. Histamine’s Role in AR Pathophysiology and AhR-Mediated Regulation

Histamine is a key effector molecule in the pathogenesis of AR, participating in both early-phase and late-phase allergic responses. Upon allergen exposure in sensitized individuals, allergens crosslink allergen-specific IgE bound to the high-affinity IgE receptor (FcεRI) on the surface of mast cells, triggering rapid mast cell degranulation and the release of preformed inflammatory mediators, among which histamine is a major component [21]. The immediate release of histamine can directly activate sensory nerve endings in the nasal mucosa, inducing neurogenic inflammatory responses manifested as typical symptoms such as sneezing and nasal itching [22].

Beyond its role in acute symptom induction, histamine can also sustain and amplify local inflammatory responses through receptor-mediated signaling pathways. Four histamine receptor subtypes (H1–H4) have been identified. The histamine H1 receptor (H1R) is an established target for the treatment of allergic symptoms, whereas the histamine H4 receptor (H4R) has been implicated in the regulation of allergic inflammation [23]. In AR, associations between histamine H4 receptor gene (*HRH4*) polymorphisms and responses to H1 antihistamines have been reported [24]. H1R signaling has been linked to inflammatory gene regulation, including nuclear factor κB (NF-κB)-dependent responses [25]. In EA.hy926 endothelial cells, desloratadine citrate disodium reduced histamine- and lipopolysaccharide (LPS)-induced interleukin-6 (IL-6) production and NF-κB nuclear translocation [26]. In LPS-stimulated macrophages, AhR interacts with specificity protein 1 (Sp1) and inhibits its LPS-induced phosphorylation, reducing histidine decarboxylase expression and histamine synthesis. This mechanism limits H1R-associated IL-6 production [27]. Its contribution to nasal inflammation and symptoms in AR has not yet been established.

### 3.2. Neuroimmune Regulation in AR: AhR Signaling and Cholinergic Pathways

Neural signaling contributes to nasal inflammation and secretion alongside immune responses [28]. In a mouse model of irritant-induced nasal inflammation, solitary chemosensory cells (SCCs) activated peptidergic trigeminal fibers through nicotinic acetylcholine receptors. Substance P signaling through neurokinin-1 receptors contributed to mast cell degranulation and plasma leakage [29]. This pathway was demonstrated outside an IgE-dependent AR model. Separately, parasympathetic cholinergic signaling stimulates nasal glandular secretion through muscarinic receptors [30]. These sensory-inflammatory and secretomotor pathways should be distinguished when considering possible effects of AhR on nasal symptoms.

Acetylcholinesterase (AChE) hydrolyzes acetylcholine and helps terminate cholinergic signaling. In human SK-N-SH neuronal cells, TCDD reduced AChE expression and activity, and CH223191 counteracted this effect, supporting AhR involvement [31]. Related inhibitory effects have been reported for other dioxin-like compounds in neuronal cultures [32]. However, the response is not uniform: TCDD did not significantly alter AChE activity or mRNA expression in contractile rat primary skeletal muscle cells [33], and astrocyte-conditioned medium modified neuronal responses according to the stage of differentiation [34]. These findings indicate that the effects of TCDD on AChE depend on the experimental system and cannot be assumed to occur in nasal nerves or glands.

**Figure 2:**
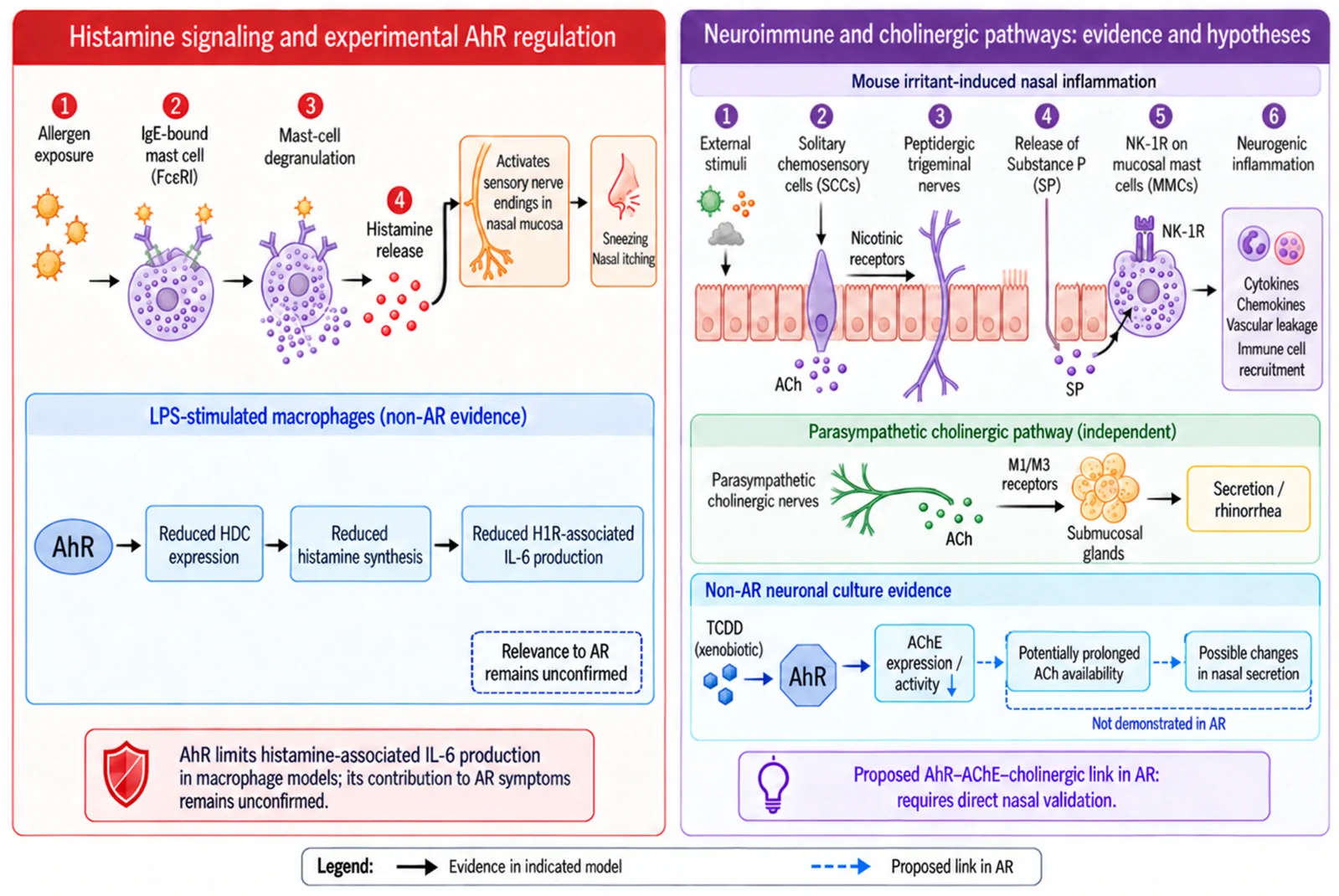
Experimental findings and proposed links between AhR signaling and nasal neuroimmune responses. The (**left**) panel separates histamine release following allergen-induced mast-cell degranulation from AhR-dependent suppression of histamine-associated IL-6 production in LPS-stimulated macrophages. The (**right**) panel distinguishes three processes: the SCC–trigeminal nerve–substance P pathway demonstrated in mouse irritant-induced nasal inflammation; parasympathetic stimulation of glandular secretion through muscarinic receptors; and TCDD-associated suppression of AChE in non-AR neuronal cultures. If comparable AChE suppression occurs in the nasal cholinergic compartment, it could prolong local ACh availability and increase glandular secretion. This complete AhR–AChE–secretion pathway has not been established in AR. Solid arrows summarize pathway or regulatory links in the indicated contexts; dashed blue arrows indicate proposed links requiring validation in AR. The downward arrow denotes reduced AChE expression or activity. The background histaminergic and neural pathways are not all established AhR-dependent mechanisms in AR. Abbreviations: ACh, acetylcholine; AChE, acetylcholinesterase; AhR, aryl hydrocarbon receptor; AR, allergic rhinitis; FcεRI, high-affinity IgE receptor; H1R, histamine H1 receptor; HDC, histidine decarboxylase; IgE, immunoglobulin E; IL-6, interleukin-6; LPS, lipopolysaccharide; M1/M3, muscarinic acetylcholine receptor subtypes 1 and 3; MMCs, mucosal mast cells; NK-1R, neurokinin-1 receptor; SCCs, solitary chemosensory cells; SP, substance P; TCDD, 2,3,7,8-tetrachlorodibenzo-p-dioxin.

A possible connection to nasal secretion can nevertheless be proposed. If AhR-dependent AChE suppression occurs near nasal cholinergic nerve terminals, reduced acetylcholine degradation could prolong its local availability and increase muscarinic receptor stimulation. Human nasal mucosal explant experiments support a role for M1 and M3 receptors in cholinergically stimulated secretion [30]. However, the cited studies do not demonstrate the complete AhR–AChE–acetylcholine–glandular secretion pathway in human AR or AR animal models. This pathway should therefore be considered a mechanistic hypothesis. Its validation would require measurements of nasal AChE activity, acetylcholine availability, and secretion following AhR manipulation, together with experiments testing whether AChE restoration or muscarinic blockade reverses the secretory effect. Figure 2 distinguishes the experimental findings from the proposed links to nasal neuroimmune responses.

## 4. AhR-Mediated Regulation of Immune Cells Relevant to AR

### 4.1. AhR Signaling in Mast Cell Regulation and Its Role in AR Pathogenesis

During sensitization, allergen presentation promotes type 2 immune responses and allergen-specific IgE production. On subsequent exposure, allergen-mediated crosslinking of IgE bound to FcεRI triggers mast cell activation and degranulation [21,35].

AhR ligands modulate mast cell responses in a manner that depends on the ligand and exposure conditions. In RBL-2H3 cells, Maaetoft-Udsen et al. reported changes in calcium signaling, degranulation, and cytokine expression after treatment with different AhR ligands; some effects could not be attributed exclusively to AhR signaling [36]. Sibilano et al. found that a single exposure to 6-formylindolo[3,2-b]carbazole (FICZ) enhanced mast cell degranulation, whereas repeated exposure attenuated this response [37]. AhR-deficient mice and mast cells also exhibit defects in mast cell development, survival, and signaling [38]. These genetic findings demonstrate a role in cellular homeostasis but should not be equated with the effects of short-term pharmacological inhibition.

In mouse and human mast cell cultures, kynurenine enhanced IgE-mediated activation, and CH223191 inhibited this enhancement [39]. Ethyl caffeate inhibited FICZ- or 1,4-dihydroxy-2-naphthoic acid (DHNA)-induced *Cyp1a1* expression and DHNA-induced IL-6 production in mouse bone marrow-derived mast cells; it also reduced mast cell degranulation and passive cutaneous anaphylaxis in the tested systems [40]. These studies provide mechanistic evidence from non-AR models. Whether comparable effects can be achieved in nasal mast cells without impairing their homeostatic functions remains to be determined.

### 4.2. AhR Regulation of B-Cell Function and Its Implications in AR

Local IgE production contributes to allergic responses in the nasal mucosa [41]. During sensitization, antigen presentation and T-cells help promote allergen-specific B-cell responses and IgE production [42]. IgE binds to FcεRI on mast cells and basophils, sensitizing these cells to subsequent allergen exposure [42]. Analyses of B-cell immunoglobulin sequences and activation-induced cytidine deaminase expression in nasal biopsies provide evidence for local somatic hypermutation, clonal expansion, and class-switch recombination in patients with AR [43]. These findings support local diversification of the IgE response, but do not by themselves establish the generation of abnormally high-affinity IgE.

AhR expression increases following B-cell activation. In mouse B-cell systems, AhR limits class-switch recombination and plasma-cell differentiation, whereas a direct role in promoting memory B-cell formation remains unresolved [44]. In these mouse B-cell systems, AhR regulates activation-induced cytidine deaminase expression and limits B lymphocyte-induced maturation protein 1 (Blimp-1) expression through the transcription factor Bach2, providing mechanistic links to class-switch recombination and plasma-cell differentiation [44]. The synthetic AhR agonist VAF347 inhibits IgE production by human B cells and suppresses dendritic cell-mediated T-cell responses; related studies establish AhR involvement in its anti-inflammatory activity [45,46]. These findings derive from cell cultures and allergic lung inflammation models rather than clinical treatment of AR. TCDD also suppresses B-cell differentiation and antibody responses, although much of this evidence comes from mouse systems [47]. Suppression of antibody production by an environmental toxicant should not, by itself, be interpreted as a therapeutically beneficial effect. The relevance of these mechanisms to local IgE production in AR therefore requires direct investigation.

### 4.3. AhR Modulation of T-Cell Differentiation and Its Role in Nasal Epithelial Barrier Integrity in AR

T-cell-derived cytokines can modify nasal epithelial barrier function by altering tight junctions and epithelial permeability [48]. Disruption of tight junctions can create paracellular leakage pathways and reduce transepithelial resistance (TER), thereby weakening mucosal barrier function and facilitating allergen penetration [48]. During allergic sensitization, dendritic cells take up inhaled allergens and present allergen-derived peptides to naïve CD4^+^ T cells, promoting T helper 2 (Th2) differentiation [48]. Th2-associated cytokines contribute to IgE production and allergic inflammation in AR [49].

Beyond the classical Th2 response, Th17 cells represent another important CD4^+^ T-cell subset involved in mucosal inflammatory regulation through secretion of IL-17A, IL-17F, and IL-22 [50]. In an AR mouse model, *Notch2* overexpression promoted regulatory T (Treg) cell differentiation and reduced allergic inflammation [51]. This supports a role for Treg-associated regulation in AR but does not establish an AhR-dependent mechanism.

The effects of AhR on Th17 responses must be interpreted within their experimental context. Studies of murine T cells under Th17-polarizing conditions and of experimental autoimmune encephalomyelitis support a role for AhR in Th17 differentiation and show that FICZ can enhance Th17-associated responses [52,53]. These are non-AR systems. In contrast, 2-(1′H-indole-3′-carbonyl)-thiazole-4-carboxylic acid methyl ester (ITE) suppressed Th17 responses in cultured dendritic cells and CD4^+^ T cells obtained from patients with AR [54]. This provides AR-related in vitro evidence, but does not establish clinical efficacy or a uniform effect of all AhR agonists.

AhR signaling can also influence epithelial barrier outcomes. In a study combining human AR nasal tissue analysis with epithelial cell experiments and an AR mouse model, PM_2.5_ exposure promoted AhR/CYP1A1/ROS signaling and NLRP3-mediated pyroptosis [14]. Experimental AhR downregulation reduced pyroptosis and barrier dysfunction. These findings support a detrimental role for AhR signaling in the specific context of pollutant-associated epithelial injury.

**Figure 3:**
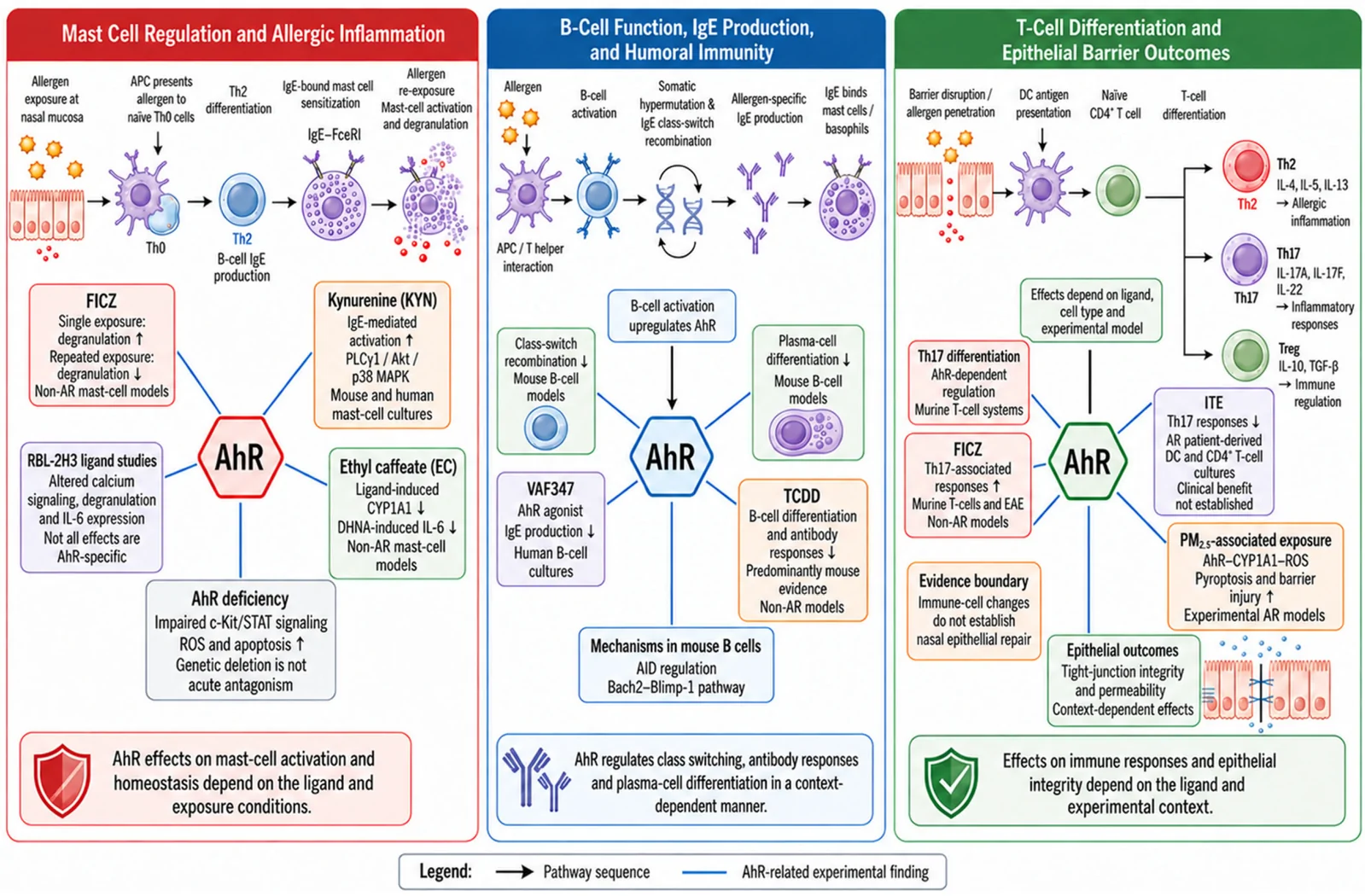
Experimental findings on AhR regulation of immune responses relevant to AR. The panels summarize ligand- and context-dependent effects on mast cells, B cells, T-cell responses, and epithelial outcomes. Model labels distinguish AR-related evidence from findings obtained in non-AR systems. Black arrows indicate pathway sequences, and plain blue lines connect AhR with the experimental findings summarized in adjacent boxes; the blue lines do not indicate activation or inhibition. Upward and downward arrows within the boxes indicate reported increases and decreases, respectively. Effects on immune-cell responses do not, by themselves, establish restoration of nasal epithelial barrier function. Abbreviations and labels: AID, activation-induced cytidine deaminase; AhR, aryl hydrocarbon receptor; Akt, protein kinase B; APC, antigen-presenting cell; AR, allergic rhinitis; Bach2, BTB and CNC homolog 2; Blimp-1, B lymphocyte-induced maturation protein 1; CD4, cluster of differentiation 4; c-Kit, stem cell factor receptor KIT; CYP1A1, cytochrome P450 1A1; DC, dendritic cell; DHNA, 1,4-dihydroxy-2-naphthoic acid; EAE, experimental autoimmune encephalomyelitis; EC, ethyl caffeate; FcεRI, high-affinity IgE receptor; FICZ, 6-formylindolo[3,2-b]carbazole; IgE, immunoglobulin E; IL, interleukin; ITE, 2-(1′H-indole-3′-carbonyl)-thiazole-4-carboxylic acid methyl ester; KYN, kynurenine; p38 MAPK, p38 mitogen-activated protein kinase; PLCγ1, phospholipase Cγ1; PM_2.5_, particulate matter with an aerodynamic diameter of 2.5 μm or less; ROS, reactive oxygen species; STAT, signal transducer and activator of transcription; TCDD, 2,3,7,8-tetrachlorodibenzo-p-dioxin; TGF-β, transforming growth factor-β; Th0, naïve helper T cells; Th2, T helper 2 cells; Th17, T helper 17 cells; Treg, regulatory T cells. RBL-2H3 denotes a rat basophilic leukemia cell line, and VAF347 is a synthetic AhR agonist.

The epithelial and T-cell findings address different outcomes and should not be treated as interchangeable evidence. In particular, suppression of Th17 responses in patient-derived immune cells does not by itself demonstrate restoration of nasal tight-junction integrity or normalization of epithelial permeability. Whether selected AhR ligands can improve both immune regulation and barrier function in AR requires studies that assess these outcomes together. Figure 3 summarizes the ligand- and model-dependent findings across immune-cell and epithelial systems.

Table 1 compares representative ligands and exposures according to experimental model, target cell type, measured outcome, and response direction.

## 5. Conclusions

Available evidence links AhR signaling to epithelial and immune responses relevant to AR. Through its interactions with diverse endogenous and exogenous ligands, AhR participates in the modulation of epithelial barrier integrity, immune cell differentiation, inflammatory mediator production, and potentially neuroimmune signaling pathways. These multifaceted roles extend the conventional Th2-centered paradigm of AR pathogenesis and underscore the complexity of mucosal immune regulation in allergic airway disease.

The evidence reviewed here indicates that AhR cannot be classified as uniformly protective or pathogenic in AR. Its effects depend on the activating ligand and the responding cell type. In experimental models of PM_2.5_-associated AR, AhR signaling contributes to nasal epithelial pyroptosis and barrier dysfunction, and AhR downregulation alleviates these changes [14]. In mast cell models, kynurenine enhances IgE-mediated activation through AhR, whereas the AhR antagonist CH223191 inhibits this enhancement [39]. Conversely, the AhR ligand ITE suppresses Th17 responses in cultured dendritic cells and CD4^+^ T cells from patients with AR [54]. The synthetic AhR agonist VAF347 also inhibits IgE production by human B cells and shows anti-inflammatory activity in experimental allergic responses [45,46]. These findings support context-dependent effects rather than a single direction of AhR action.

Accordingly, neither general AhR blockade nor indiscriminate activation with a synthetic agonist can currently be recommended for AR on the basis of the evidence reviewed here. AhR inhibition may warrant investigation in pollutant-exposed nasal epithelial cells and in mast cells showing ligand-enhanced activation [14,39]. Conversely, selective activation may warrant investigation in dendritic cells and CD4^+^ T cells to suppress Th17 responses, or in B cells to reduce IgE production, as suggested by studies of ITE and the synthetic AhR agonist VAF347, respectively [45,46,54]. Future studies should therefore evaluate individual ligands in defined cellular contexts and determine whether their beneficial effects can be achieved without enhancing pro-allergic responses in other cell populations.

## Figures and Tables

**Table 1:** Context-dependent effects of selected AhR ligands and related exposures.

Ligand or Exposure	Experimental Model and Relationship to AR	Target Cells	Measured Outcomes and Response Direction	References
PM_2.5_-associated exposure *	Human AR nasal tissue observations; epithelial cell experiments; AR mouse model	Nasal epithelial cells	Increased pyroptosis and barrier dysfunction in exposure models; AhR downregulation attenuated injury	[14]
ITE	Cultured cells from patients with AR; AR-related in vitro evidence	Dendritic cells and CD4^+^ T cells	Reduced Th17 responses and increased IL-10 production	[54]
FICZ	Mast cell cultures and mouse passive systemic anaphylaxis; non-AR models	Mast cells	Single exposure enhanced degranulation and histamine release; repeated exposure attenuated these responses	[37]
FICZ	Murine T-cell experiments and experimental autoimmune encephalomyelitis; non-AR models	CD4^+^ T cells	Enhanced Th17-associated responses under the tested conditions	[52]
Kynurenine	Mouse bone marrow-derived and human cultured mast cells; mouse cutaneous anaphylaxis; non-AR models	Mast cells	Enhanced IgE-mediated activation; CH223191 inhibited the enhancement	[39]
VAF347	Human cell cultures and mouse allergic lung inflammation; non-AR models	B cells and dendritic cells	Reduced IgE production and dendritic cell-mediated T-cell activation; reduced allergic lung inflammation	[45,46]
TCDD	Human SK-N-SH neuronal cell culture; non-AR model	Neuronal cells	Reduced AChE expression and activity; nasal secretion was not assessed	[31]
TCDD	Contractile rat primary skeletal muscle culture; non-AR model	Differentiating skeletal muscle cells	No significant change in AChE activity or mRNA expression under the tested conditions	[33]
Ethyl caffeate	Mouse mast cell cultures and passive cutaneous anaphylaxis; non-AR models	Mast cells	Inhibited FICZ/DHNA-induced AhR signaling and mast cell activation-related outcomes	[40]

* PM_2.5_ is a complex environmental exposure, not a single chemically defined AhR ligand. Response directions apply to the specified models and exposure conditions and should not be generalized to all AR contexts. Abbreviations: AChE, acetylcholinesterase; AhR, aryl hydrocarbon receptor; AR, allergic rhinitis; CD4, cluster of differentiation 4; DHNA, 1,4-dihydroxy-2-naphthoic acid; FICZ, 6-formylindolo[3,2-b]carbazole; IgE, immunoglobulin E; IL-10, interleukin-10; ITE, 2-(1′H-indole-3′-carbonyl)-thiazole-4-carboxylic acid methyl ester; mRNA, messenger RNA; PM_2.5_, particulate matter with an aerodynamic diameter of 2.5 μm or less; TCDD, 2,3,7,8-tetrachlorodibenzo-p-dioxin; Th17, T helper 17 cells. CH223191 denotes an AhR antagonist, and VAF347 denotes a synthetic AhR agonist.

## Data Availability

No new data were created or analyzed in this study. Data sharing is not applicable to this article.

## References

[1] Skoner D.P. (2001). Allergic rhinitis: Definition, epidemiology, pathophysiology, detection, and diagnosis. J. Allergy Clin. Immunol..

[2] Bousquet J., Anto J.M., Bachert C., Baiardini I., Bosnic-Anticevich S., Walter Canonica G., Melén E., Palomares O., Scadding G.K., Togias A. (2020). Allergic rhinitis. Nat. Rev. Dis. Primers.

[3] Zhang Y., Lan F., Zhang L. (2021). Advances and highlights in allergic rhinitis. Allergy.

[4] Wise S.K., Damask C., Roland L.T., Ebert C., Levy J.M., Lin S., Luong A., Rodriguez K., Sedaghat A.R., Toskala E. (2023). International consensus statement on allergy and rhinology: Allergic rhinitis—2023. Int. Forum Allergy Rhinol..

[5] Savouré M., Bousquet J., Jaakkola J.J.K., Jaakkola M.S., Jacquemin B., Nadif R. (2022). Worldwide prevalence of rhinitis in adults: A review of definitions and temporal evolution. Clin. Transl. Allergy.

[6] Ferguson B.J. (2004). Influences of allergic rhinitis on sleep. Otolaryngol.-Head Neck Surg..

[7] Yang T., Wang H.R., Mou Y.K., Liu W.C., Wang Y., Song X.Y., Ren C., Song X.C. (2024). Mutual Influence Between Allergic Rhinitis and Sleep: Factors, Mechanisms, and interventions-A Narrative Review. Nat. Sci. Sleep.

[8] Bousquet J., Khaltaev N., Cruz A.A., Denburg J., Fokkens W.J., Togias A., Zuberbier T., Baena-Cagnani C.E., Canonica G.W., van Weel C. (2008). Allergic Rhinitis and its Impact on Asthma (ARIA) 2008 update (in collaboration with the World Health Organization, GA(2)LEN and AllerGen). Allergy.

[9] Wang J., Zhou Y., Zhang H., Hu L., Liu J., Wang L., Wang T., Zhang H., Cong L., Wang Q. (2023). Pathogenesis of allergic diseases and implications for therapeutic interventions. Signal Transduct. Target. Ther..

[10] Cella M., Colonna M. (2015). Aryl hydrocarbon receptor: Linking environment to immunity. Semin Immunol..

[11] Denison M.S., Nagy S.R. (2003). Activation of the aryl hydrocarbon receptor by structurally diverse exogenous and endogenous chemicals. Annu Rev. Pharmacol. Toxicol..

[12] Safe S., Jin U.H., Park H., Chapkin R.S., Jayaraman A. (2020). Aryl Hydrocarbon Receptor (AHR) Ligands as Selective AHR Modulators (SAhRMs). Int. J. Mol. Sci..

[13] Gutiérrez-Vázquez C., Quintana F.J. (2018). Regulation of the Immune Response by the Aryl Hydrocarbon Receptor. Immunity.

[14] Yuan J., Liao Z., Zhu X., Zhu Y., Wu S., Guo L., Fu Y., Liu Y. (2025). PM(2.5) exacerbates nasal epithelial barrier dysfunction in allergic rhinitis by inducing NLRP3-mediated pyroptosis via the AhR/CYP1A1/ROS axis. J. Hazard Mater..

[15] Riaz F., Pan F., Wei P. (2022). Aryl hydrocarbon receptor: The master regulator of immune responses in allergic diseases. Front Immunol..

[16] Neavin D.R., Liu D., Ray B., Weinshilboum R.M. (2018). The Role of the Aryl Hydrocarbon Receptor (AHR) in Immune and Inflammatory Diseases. Int. J. Mol. Sci..

[17] Wei P., Hu G.H., Kang H.Y., Yao H.B., Kou W., Liu H., Hong S.L. (2014). Increased aryl hydrocarbon receptor expression in patients with allergic rhinitis. QJM.

[18] Stockinger B., Di Meglio P., Gialitakis M., Duarte J.H. (2014). The aryl hydrocarbon receptor: Multitasking in the immune system. Annu Rev. Immunol..

[19] Marshall N.B., Kerkvliet N.I. (2010). Dioxin and immune regulation: Emerging role of aryl hydrocarbon receptor in the generation of regulatory T cells. Ann. N. Y. Acad. Sci..

[20] Chen L., Xiao L., Liu J., Shen Y., Ke X., Huang J., Hu G., Yang Y. (2017). Differential Expression of the Aryl Hydrocarbon Receptor and Transforming Growth Factor Beta 1 in Chronic Rhinosinusitis with Nasal Polyps with Allergic Rhinitis. ORL J. Otorhinolaryngol. Relat. Spec..

[21] Galli S.J., Tsai M., Piliponsky A.M. (2008). The development of allergic inflammation. Nature.

[22] Okubo K., Kurono Y., Ichimura K., Enomoto T., Okamoto Y., Kawauchi H., Suzaki H., Fujieda S., Masuyama K. (2017). Japanese guidelines for allergic rhinitis 2017. Allergol. Int..

[23] Thurmond R.L., Gelfand E.W., Dunford P.J. (2008). The role of histamine H1 and H4 receptors in allergic inflammation: The search for new antihistamines. Nat. Rev. Drug Discov..

[24] Gu J., Mao X.H., Yang X.Z., Ao H.F., Zhang Z., Li Y. (2017). Histamine H4 receptor gene polymorphisms: A potential predictor of oral H1 antihistamine efficacy for allergic rhinitis. Int. Forum Allergy Rhinol..

[25] Leurs R., Church M.K., Taglialatela M. (2002). H1-antihistamines: Inverse agonism, anti-inflammatory actions and cardiac effects. Clin. Exp. Allergy.

[26] Jie Q., Kodithuwakku N.D., Yuan X., He G., Chen M., Xu S., Wu Y. (2015). Anti-allergic and anti-inflammatory properties of a potent histamine H1 receptor antagonist, desloratadine citrate disodium injection, and its anti-inflammatory mechanism on EA.hy926 endothelial cells. Eur. J. Pharmacol..

[27] Masuda K., Kimura A., Hanieh H., Nguyen N.T., Nakahama T., Chinen I., Otoyo Y., Murotani T., Yamatodani A., Kishimoto T. (2011). Aryl hydrocarbon receptor negatively regulates LPS-induced IL-6 production through suppression of histamine production in macrophages. Int. Immunol..

[28] Klimov V., Cherevko N., Klimov A., Novikov P. (2022). Neuronal-Immune Cell Units in Allergic Inflammation in the Nose. Int. J. Mol. Sci..

[29] Saunders C.J., Christensen M., Finger T.E., Tizzano M. (2014). Cholinergic neurotransmission links solitary chemosensory cells to nasal inflammation. Proc. Natl. Acad. Sci. USA.

[30] Mullol J., Baraniuk J.N., Logun C., Mérida M., Hausfeld J., Shelhamer J.H., Kaliner M.A. (1992). M1 and M3 muscarinic antagonists inhibit human nasal glandular secretion in vitro. J. Appl. Physiol. (1985).

[31] Xie H.Q., Xu H.M., Fu H.L., Hu Q., Tian W.J., Pei X.H., Zhao B. (2013). AhR-mediated effects of dioxin on neuronal acetylcholinesterase expression in vitro. Environ. Health Perspect..

[32] Xu H.M., Xie H.Q., Tao W.Q., Zhou Z.G., Li S.Z., Zhao B. (2014). Dioxin and dioxin-like compounds suppress acetylcholinesterase activity via transcriptional downregulations in vitro. J. Mol. Neurosci..

[33] Luo Y., Xie H.Q., Chen Y., Xia Y., Sha R., Liu Y., Ma Y., Xu T., Xu L., Wah-Keung Tsim K. (2019). Effect of 2,3,7,8-tetrachlorodibenzo-p-dioxin exposure on acetylcholinesterase during myogenic differentiation of contractile rat primary skeletal muscle cells. Chem. Biol. Interact..

[34] Sha R., Chen Y., Luo Y., Liu Y., Xu L., Xie H.Q., Zhao B. (2019). Effects of astrocyte conditioned medium on neuronal AChE expression upon 2,3,7,8-tetrachlorodibenzo-p-dioxin exposure. Chem. Biol. Interact..

[35] Erjefält J.S. (2014). Mast cells in human airways: The culprit?. Eur. Respir. Rev..

[36] Maaetoft-Udsen K., Shimoda L.M., Frøkiær H., Turner H. (2012). Aryl hydrocarbon receptor ligand effects in RBL2H3 cells. J. Immunotoxicol..

[37] Sibilano R., Frossi B., Calvaruso M., Danelli L., Betto E., Dall’Agnese A., Tripodo C., Colombo M.P., Pucillo C.E., Gri G. (2012). The aryl hydrocarbon receptor modulates acute and late mast cell responses. J. Immunol..

[38] Zhou Y., Tung H.Y., Tsai Y.M., Hsu S.C., Chang H.W., Kawasaki H., Tseng H.C., Plunkett B., Gao P., Hung C.H. (2013). Aryl hydrocarbon receptor controls murine mast cell homeostasis. Blood.

[39] Kawasaki H., Chang H.W., Tseng H.C., Hsu S.C., Yang S.J., Hung C.H., Zhou Y., Huang S.K. (2014). A tryptophan metabolite, kynurenine, promotes mast cell activation through aryl hydrocarbon receptor. Allergy.

[40] Nguyen P.T., Nakamura Y., Tran N.Q.V., Ishimaru K., Nguyen T.A., Kobayashi Y., Watanabe-Saito F., Okuda T., Nakano N., Nakao A. (2023). Ethyl Caffeate Can Inhibit Aryl Hydrocarbon Receptor (AhR) Signaling and AhR-Mediated Potentiation of Mast Cell Activation. Int. J. Mol. Sci..

[41] Pawankar R., Yamagishi S., Yagi T. (2000). Revisiting the roles of mast cells in allergic rhinitis and its relation to local IgE synthesis. Am. J. Rhinol..

[42] Pointner L.N., Ferreira F., Aglas L. (2022). B Cell Functions in the Development of Type I Allergy and Induction of Immune Tolerance. Handb. Exp. Pharmacol..

[43] Coker H.A., Durham S.R., Gould H.J. (2003). Local somatic hypermutation and class switch recombination in the nasal mucosa of allergic rhinitis patients. J. Immunol..

[44] Vaidyanathan B., Chaudhry A., Yewdell W.T., Angeletti D., Yen W.F., Wheatley A.K., Bradfield C.A., McDermott A.B., Yewdell J.W., Rudensky A.Y. (2017). The aryl hydrocarbon receptor controls cell-fate decisions in B cells. J. Exp. Med..

[45] Lawrence B.P., Denison M.S., Novak H., Vorderstrasse B.A., Harrer N., Neruda W., Reichel C., Woisetschläger M. (2008). Activation of the aryl hydrocarbon receptor is essential for mediating the anti-inflammatory effects of a novel low-molecular-weight compound. Blood.

[46] Ettmayer P., Mayer P., Kalthoff F., Neruda W., Harrer N., Hartmann G., Epstein M.M., Brinkmann V., Heusser C., Woisetschläger M. (2006). A novel low molecular weight inhibitor of dendritic cells and B cells blocks allergic inflammation. Am. J. Respir. Crit. Care Med..

[47] Sulentic C.E., Kaminski N.E. (2011). The long winding road toward understanding the molecular mechanisms for B-cell suppression by 2,3,7,8-tetrachlorodibenzo-p-dioxin. Toxicol. Sci..

[48] Yang M., Sun L., Zhu D., Meng C., Sha J. (2023). Recent advances in understanding the effects of T lymphocytes on mucosal barrier function in allergic rhinitis. Front Immunol..

[49] Small P., Kim H. (2011). Allergic rhinitis. Allergy Asthma Clin. Immunol..

[50] Miossec P., Kolls J.K. (2012). Targeting IL-17 and TH17 cells in chronic inflammation. Nat. Rev. Drug Discov..

[51] Jiao W.E., Sun L., Xu S., Deng Y.Q., Qiao Y.L., Xi Y., Tao Z.Z., Chen S.M. (2021). Notch2 suppresses the development of allergic rhinitis by promoting FOXP3 expression and Treg cell differentiation. Life Sci..

[52] Veldhoen M., Hirota K., Westendorf A.M., Buer J., Dumoutier L., Renauld J.C., Stockinger B. (2008). The aryl hydrocarbon receptor links TH17-cell-mediated autoimmunity to environmental toxins. Nature.

[53] Kimura A., Naka T., Nohara K., Fujii-Kuriyama Y., Kishimoto T. (2008). Aryl hydrocarbon receptor regulates Stat1 activation and participates in the development of Th17 cells. Proc. Natl. Acad. Sci. USA.

[54] Wei P., Hu G.H., Kang H.Y., Yao H.B., Kou W., Liu H., Zhang C., Hong S.L. (2014). An aryl hydrocarbon receptor ligand acts on dendritic cells and T cells to suppress the Th17 response in allergic rhinitis patients. Lab. Investig..

